# Evolution of β-Lactam Antibiotic Resistance in *Proteus* Species: From Extended-Spectrum and Plasmid-Mediated AmpC β-Lactamases to Carbapenemases

**DOI:** 10.3390/microorganisms13030508

**Published:** 2025-02-25

**Authors:** Branka Bedenić, Mladen Pospišil, Marina Nađ, Daniela Bandić Pavlović

**Affiliations:** 1 Biomedical Research Center Šalata, University of Zagreb School of Medicine, Department for Clinical Microbiology and Infection Prevention and Control, University Hospital Centre Zagreb, 10000 Zagreb, Croatia; 2 Department of Emergency Medicine, University Hospital Centre Zagreb, 10000 Zagreb, Croatia; mladenpospisil@gmail.com; 3 University of Zagreb School of Medicine, 10000 Zagreb, Croatia; mnad@student.mef.hr; 4 Department of Anesthesiology and Intensive Care, University of Zagreb School of Medicine, University Hospital Centre Zagreb, 10000 Zagreb, Croatia; dani.bandic@gmail.com

**Keywords:** *Proteus mirabilis*, extended-spectrum β-lactamases, plasmid-mediated AmpC β-lactamases, carbapenemases, fluoroquinolone resistance

## Abstract

The management of infectious diseases has proven to be a daunting task for clinicians worldwide, and the rapid development of antibiotic resistance among Gram-negative bacteria is making it even more challenging. The first-line therapy is empirical, and it most often comprises β-lactam antibiotics. Among Gram-negative bacteria, *Proteus mirabilis*, an important community and hospital pathogen associated primarily with urinary tract and wound infection, holds a special place. This review’s aim was to collate and examine recent studies investigating β-lactam resistance phenotypes and mechanisms of *Proteus* species and the global significance of its β-lactam resistance evolution. Moreover, the genetic background of resistance traits and the role of mobile genetic elements in the dissemination of resistance genes were evaluated. *P. mirabilis* as the dominant pathogen develops resistance to expanded-spectrum cephalosporins (ESC) by producing extended-spectrum β-lactamases (ESBL) and plasmid-mediated AmpC β-lactamases (p-AmpC). β-lactamase-mediated resistance to carbapenems in *Enterobacterales*, including *Proteus* spp., is mostly due to expression of carbapenemases of class A (KPC); class B (metallo-β-lactamases or MBLs of IMP, VIM, or NDM series); or class D or carbapenem-hydrolyzing oxacillinases (CHDL). Previously, a dominant ESBL type in *P. mirabilis* was TEM-52; yet, lately, it has been replaced by CTX-M variants, particularly CTX-M-14. ESC resistance can also be mediated by p-AmpC, with CMY-16 as the dominant variant. Carbapenem resistance in *Proteus* spp. is a challenge due to its intrinsic resistance to colistin and tigecyclin. The first carbapenemases reported belonged to class B, most frequently VIM-1 and NDM-5. In Europe, predominantly France and Belgium, a clonal lineage positive for OXA-23 CHDL spreads rapidly undetected, due to its low-level resistance to carbapenems. The amazing capacity of *Proteus* spp. to accumulate a plethora of various resistance traits is leading to multidrug or extensively drug-resistant phenotypes.

## 1. Introduction

The rapid emergence of antibiotic resistance among Gram-negative bacteria is a serious threat to the management of infectious diseases. β-lactam antibiotics are the most frequently used antimicrobials for empirical therapy [1,2]. Production of β-lactamases is one of the strategies adopted by bacteria to develop resistance to β-lactam class of antibiotics [3].

*Proteus mirabilis* is an important community and hospital pathogen. It is associated primarily with urinary tract and wound infections [4]. It is one of the most important causative agents of catheter-associated urinary tract infection, which can progress to urosepsis, due to its ability to form biofilm. *P. mirabilis* develops resistance to expanded-spectrum cephalosporins (ESC) by production of extended-spectrum β-lactamases (ESBLs) and plasmid-mediated AmpC β-lactamases (p-AmpC) [4]. Carbapenems are the antibiotics of choice for the treatment of infections associated with ESC-resistant isolates. In the last decades, resistance to this last line of antibiotics was reported among *Enterobacterales*, the *Proteus* spp. included.

ESBLs hydrolyze penicillin, ESC, and monobactams. They belong predominantly to three major families: TEM, SHV, and CTX-M, with rare types such as VEB, PER, and IBC mainly concentrated in some geographic regions [5]. Plasmids encoding ESBLs often carry resistance genes for non-β-lactam antibiotics such as aminoglycosides, tetracyclines, sulphonamides, chloramphenicol, and fluoroquinolones. The CTX-M family are cefotaximases, which preferentially hydrolyze cefotaxime and are native ESBLs derived from the chromosomal β-lactamases of bacteria belonging to the genus *Kluyvera*, unlike TEM or SHV variants, which are derived by point mutations from parenthal broad-spectrum TEM-1, TEM-2, and SHV-1 β-lactamases [6]. Five phylogenetic clonal lineages of CTX-M β-lactamases exist: CTX-M-1, CTX-M-2, CTX-M-8, CTX-M-9, and CTX-M-25 [7]. At the beginning of ESBL spread in the 1980s, the TEM and SHV variants were dominant, but now, they are outnumbered by the members of the CTX-M family, particularly the CTX-M-15 variant, originally described in India, which efficiently hydrolyzes ceftazidime and cefepime as well, unlike other CTX-M types [8].

AmpC β-lactamases are primarily cephalosporinases encoded by chromosomes or plasmids. Plasmid-mediated AmpC β-lactamases (p-AmpC) are derived from the chromosomally encoded enzymes of organisms such as *Enterobacter cloacae*, *Citrobacter freundii*, and *Morganella morganii*. These enzymes have been detected in *Escherichia coli*, *Klebsiella pneumoniae*, *Salmonella* spp., and *P. mirabilis*, which are species without the chromosomal *amp*C gene or it is not expressed, like in *Escherichia coli* [9]. Those β-lactamases confer resistance to first-, second-, and third-generation cephalosporins, monobactams, and β-lactam–inhibitor combinations but spare cefepime and carbapenems.

Carbapenems are the antibiotics of choice for the treatment of infections due to ESBL- or p-AmpC-producing *Proteus* spp. isolates. β-lactamase-mediated resistance to carbapenems in Enterobacterales including *Proteus* spp. is mostly due to the expression of carbapenemases of class A (KPC), class B (metallo-β-lactamases or MBLs of IMP VIM or NDM series), or class D (OXA-48) [10]. However, porin loss or upregulation of efflux pumps can contribute to resistance [10,11]. Increased resistance can also be a consequence of reduced expression of PBP-1 and PBP-2 [12].

Resistance to aminoglycosides is mediated by the production of acetyltransferases, adenytransferases, and phosphorylases, which modify aminoglycosides and render them inactive. They are encoded by plasmid-mediated *aac*(3), *aac*(6), *aad*1, *aad*2, and *aph*(3) genes. Panaminoglycoside resistance is associated with *rmt* and *arm* genes, which encode 16S rRNA methylase, protecting the ribosomal receptor. Fluoroquinolone resistance in *Proteus* species is attributed to the mutations of the chromosomal *gyr*A and *par*C genes and the acquisition of plasmid-mediated *qnr* genes, which encode qnr proteins, protecting DNA gyrase [13].

Reports on antibiotic resistance in *P. mirabilis* and *Proteus vulgaris* as two species with particular clinical importance are still scarce in the medical bibliography. In this review, we analyzed the evolution of antibiotic resistance in *Proteus* spp., the genetic background of β-lactam resistance determinants, the role of mobile genetic elements in the dissemination of resistance genes, laboratory detection of resistance traits, and clinical importance of antimicrobial resistance.

## 2. Intrinsic Resistance

*Proteus* spp. are naturally resistant to several antibiotics, including colistin and nitrofurantoin, and exhibit reduced susceptibility to imipenem [12]. They do not produce any chromosomal β-lactamases, and thus, there is no intrinsic resistance to β-lactam antibiotics. All β-lactam resistance determinants are acquired.

## 3. Broad-Spectrum β-Lactamases

The Class A broad-spectrum clavulanic acid inhibited β-lactamases, which hydrolyze penicillin, present in *Proteus* species are TEM-1, TEM-2, and SHV-1 [12]. These isolates are recognized in routine diagnostics based on resistance to ampicillin and amoxycillin. Oxacillinases, which preferentially hydrolyze oxacillin, reported among *Proteus* species mostly from Turkey, are OXA-1, OXA-9, and OXA-10. They are not susceptible to inhibition by clavulanic acid, sulbactam, and tazobactam [14]. The newest variant OXA-320 is integron-associated [14], containing aminoglycoside resistance gene *aad*1 for adenyltransferase, which modifies aminoglycosides and renders them inactive.

## 4. Extended-Spectrum β-Lactamases

Early studies found TEM-52 ESBLs to be the dominant resistance determinants to ESC in *P. mirabilis* [15,16,17,18]. TEM-52 was found in *P. mirabilis* from the Southern Mediterranean region of Croatia [15,16] and Italy [17,18]. TEM-52 is a ceftazidimase, which preferentially hydrolyzes ceftazidime. Except TEM-52, there were other TEM variants identified in *P. mirabilis* but only sporadically: TEM-11 in Hong Kong [19], TEM-15 and TEM-20 in Italy [20], and TEM-21 in France [21,22]. The majority of TEM allelic variants are ceftazidimases, which preferentially hydrolyze ceftazidime, as shown in Table 1.

SHV variants are less frequent compared to TEM and CTX-M. A hospital outbreak associated with SHV-5-producing *P. mirabilis* was reported in 2011 in Brazil [23]. SHV-5, first reported in *K. pneumoniae*, is a typical ceftazidime conferring on the producing isolates’ high level of ceftazidime resistance, with minimum inhibitory concentrations (MICs) exceeding 128 mg/L (Table 1).

A switch from TEM and SHV to CTX-M variants was observed in the early 2010s. *bla*_CTX-M_ genes in *P. mirabilis* usually generate CTX-M-2, CTX-M-3, CTX-M-14, and CTX-M-27, as illustrated in Table 1 [12,24]. CTX-M-15, which is dominant among *E. coli* and *K. pneumoniae*, is less frequent in *P. mirabilis* compared to other CTX-M variants, except in Russia [25]. *bla*_CTX-M-15_ genes are usually preceded by an insertion element IS*Ecp* responsible for the mobilization of the gene and increased gene expression, leading to high-level resistance to all cephalosporins [25]. In Tunisia, *bla*_CTX-M-15_ genes found in ESBL-positive *P. mirabilis* were chromosomally encoded [26]. IS*Ecp* may mediate chromosomal incorporation of the gene. CTX-M-8 is the rarest variant encountered only in Brazil [27] (Table 1). CTX-M-14 is the most widespread variant described among *Proteus* spp., first reported in this species in South Korea [28]. The isolates coharbored TEM-1, SHV-1, and OXA-10 broad-spectrum β-lactamases. Later, this variant was found in China alongside with the mutant variant CTX-M-140, which decreased hydrolytic activity, both belonging to phylogenetic group CTX-M-9 [29].

The new CTX-M-encoding gene in *P. mirabilis* is *bla*_CTX-M-65_ [30]. The isolate producing CTX-M-65 was identified in Russia with additional *bla*_VEB_-encoding Vietnam extended-spectrum β-lactamase (VEB), the *aac*6-Ib gene encoding aminoglycoside resistance, and *qnr*A1 for fluoroquinolone resistance [30]. Whole-genome sequencing (WGS) revealed that isolates belonged to two different clones. The same allelic variant that was identified in animal *P. mirabilis* isolates from Hong Kong was located in *Tn*7-like composite transposon and associated with extensively drug-resistant phenotypes [30]. Unlike previous studies, the ESBL-encoding genes were chromosomally encoded. The genes responsible for sulfonamide resistance, *sul*1 and *sul*2, and chloramphenicol *cat*B3 were located on the chromosome as well [30]. *bla*_CTX-M_ genes in *P. mirabilis* are usually carried in IncT, IncW, IncFIA, IncFIB, and IncK plasmids [12].

Rare type ESBLs such as PER and VEB are more frequent in *Proteus* spp. compared to other *Enterobacterales.* In North Africa, isolates positive for PER-1 (Pseudomonas extended resistance), originally reported in *Pseudomonas aeruginosa*, were identified in *P. vulgaris* [31]. The isolates showed high-level resistance to penicillin, ceftazidime, and aztreonam. In Europe, PER-1 was identified in France [32] and Italy [33].

Another rare type of ESBL found in Austria in *P. mirabilis* is VEB-1 (Vietnam extended-spectrum β-lactamase), previously reported in *Pseudomonas aeruginosa*, which coproduced NDM-5 MBL [34], as shown in Table 1. The patient with subphrenic abscess was previously treated with broad-spectrum cephalosporins in Bangladesh [34]. In France, VEB-6 was described in *P. mirabilis* [35]. The Class 1 integron-carrying *bla*_VEB-6_ gene contained aminoglycoside (*aac*A4, *aad*A2), trimethoprim (*dfr*A1), sulfonamide (*sul*1), tetracycline (*tet*), and fluoroquinolone (*qnr*A1) resistance genes.

ESBL positivity in *P. mirabilis* is often accompanied by fluoroquinolone resistance. High-level fluoroquinolone resistance in *P. mirabilis* is usually mediated by mutations in the *gyr*A and *par*C genes. Low-level resistance is, in most cases, associated with plasmid-mediated *qnr* genes (*qnr*A, *qnr*B, *qn*rC, *qnr*D, and *qnr*S), which generate qnr proteins to protect DNA gyrase. However, recently, *qnrA*6 was found to be chromosomally encoded in *P. mirabilis* [36]. Moreover, plasmids encoding ESBLs very frequently contain genes encoding aminoglycoside resistance: *aad*1 and *aad*2 genes encoding adenyltransferases, *aac*3Ia and *aac*6-Ib for acetyltransferases, and *aph*(3)Ia and *aph*(6)-Id responsible for phosporylases, which modify aminoglycosides to render them inactive [12]. Furthermore, the genes for sulfonamide resistance (*sul*1 and *sul*2), trimethoprim resistance encoding dyhydropholate reductase (*dfr*A1 and *drf*A32), *cat* and *cat*1 generating chloramphenicol acetyltransferase, *tet* for tetracycline resistance, and *msr* for macrolide resistance usually accompany genes for ESBLs belonging to the CTX-M family [12].

**Figure 1:**
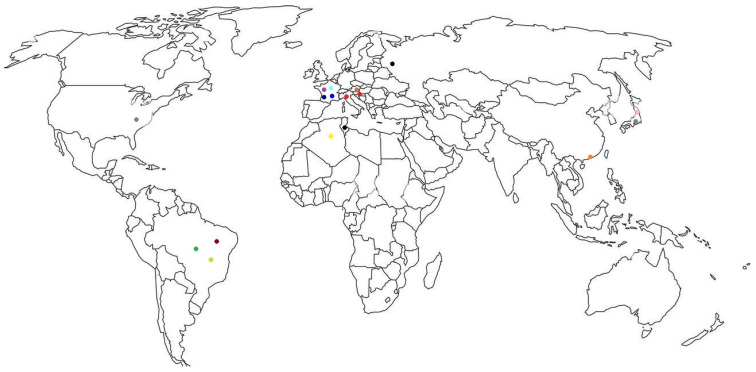
Distribution of ESBLs. TEM-11, light blue; TEM-15, blue; TEM-20, dark blue; TEM-52, red; SHV-5, dark red; CTX-M-2, light green; CTX-M-3, purple; CTX-M-8, green; CTX-M-14, gray; CTX-M-15, black; CTX-M-27, pink; CTX-M-65, orange; PER-1, yellow; VEB-1, brown.

Geographic distribution of the ESBLs is shown in Figure 1.

**Table 1:** ESBLs reported in *Proteus* species.

	Phenotype of Resistance						
Type of ESBL	CAZ	CTX	CRO	FEP	AMT	Geographic Distribution	References
TEM-11	R	R	R	R	R	France	[21,22]
TEM-15	R	R	R	R	R	France	[21,22]
TEM-20	R	R	R	R	R	France	[21,22]
TEM-52	I-R (8–>128)	R (8–128)	R (8–128)	S-R (2–32)	S-I (1–16)	Croatia Italy	[15,16,17,18]
SHV-5	R	I	I	S	R	Brazil	[23]
CTX-M-2	R	I	I	R	R	Brazil	[12]
CTX-M-3	R	I	I	R	R	France	[12]
CTX-M-8	R	I	I	R	R	Brazil	[27]
CTX-M-14	R	I	I	R	R	Japan USA	[28,29]
CTX-M-15	R	R	R	R	R	Russia, Tunisia	[25,26]
CTX-M-27	R	R	R	R	R	Japan	[24]
CTX-M-65	R	R	R	R	R	Hong-Kong	[30]
PER-1	R (>128)	R (16–32)	R	I-R (8–32)	R	Algeria	[31,32,33]
VEB-1	R	R	R	R	R	Austria France	[34,35]

Abbreviations: CAZ—ceftazidime, CTX—cefotaxime, CRO—ceftriaxone, FEP—cefepime, AMT—aztreonam, R—resistant, I—intermediate susceptible (increased exposure), and S—susceptible. The MICs (mg/L) were provided in parentheses if available in the references.

## 5. Inhibitor-Resistant β-Lactamases

Class A inhibitor-resistant variants belonging to the TEM and OXA families have also been found among *Proteus* spp. isolates [21]. The clinical significance is limited, as the isolates are susceptible to cephalosporin, where there is a plethora of various inhibitor-resistant TEM variants (IRT), derived by point mutations from TEM-1 and TEM-2 found among *Proteus* spp. isolates: TEM-65, TEM-67, TEM-73, and TEM-74, in line with the majority of reports from France [22]. The clinical relevance of IRT is limited, because the isolates are susceptible to cephalosporins.

## 6. AmpC β-Lactamases

Reports on p-AmpC β-lactamases are in the medical literature. Most publications report β-lactamases belonging to the CMY family [37]. The *Proteus* species does not have chromosomal AmpC genes, and thus, all AmpC β-lactamases are supposed to be plasmid-mediated. However, some studies proved the chromosomal integration of *bla*_ampC_ genes mediated by the IS*Ecp*1 insertion element [37].

**Figure 2:**
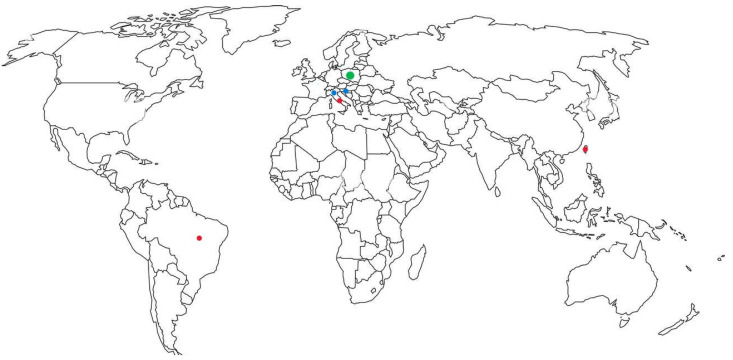
Distribution of AmpC β-lactamases in *Proteus* species. CMY-16, blue; CMY-2, red; DHA-1 green.

The acquired *bla*_CMY_ genes have escaped from the chromosome of *C. freundii* following mobilization mediated by IS*Ecp*1, IS26, or ISCR1. CMY-1, CMY-12, and CMY-16 were found to be the most prevalent variants of plasmid-mediated AmpC β-lactamases in Europe [37], as shown in Table 2. In addition, mobile insertion sequences such as IS*26* and/or IS*Ecp*1, which can be found upstream of *bla*_AmpC_ genes, can facilitate their mobilization and increase expression of the gene and the level of resistance. The simultaneous production of ESBLs and AmpC β-lactamases was also reported in *P. mirabilis* in recent studies [38]. CMY-16 was previously reported in *P. mirabilis* from a long-term care facility in Italy [39], from a nursing home in Zagreb [40], and from a hospital in Split in Croatia [41], as illustrated in Table 2. In the Italian study, TEM-92, which is an ESBL, and p-AmpC β-lactamase CMY-16 were simultaneously found [39]. In a Croatian study, the isolates from Zagreb and Split exhibited similar resistance patterns, including resistance to sulfonamides, fluoroquinolones, and to aminoglycosides, in addition to cefoxitin and ESC resistance. All isolates were susceptible to carbapenems, ceftazidime-avibactam, and Fosfomycin. *bla*_CMY_ genes were associated with an IS*Ecp* insertion element 110 bp upstream of the *bla*_CMY-16_ starting codon [41]. The isolates from Split were allocated into four clusters, as demonstrated by pulsed-field gel electrophoresis (PFGE) [41]. CMY-2 was identified in Italy [37], Taiwan [42], and Brazil [43]. It was encoded on IncA/C plasmid. DHA-1, originally described in *K. pneumoniae*, was identified among *Proteus vulgaris* isolates in Poland [44] (Table 2). It is derived from the chromosomal ampC gene of *Morganella morganii*. A DHA-encoding gene was embedded in a class 1 integron. Unlike CMY, which is constitutive, DHA variants are inducible [44]. Class 1 integrons carrying DHA genes contained *aadA*1; *aadB1*, conferring aminoglycoside resistance; *dfr*A1 for dihydrofolate reductase associated with trimethoprim resistance; and *bla*(_PSE-1_)*-aad*A1 and *aac*A4-orfA-orfB-*aad*A1 gene cassettes [44]. The geographic distribution of p-AmpC is illustrated in Figure 2.

## 7. Carbapenemases

*P. mirabilis*, similar to other Enterobacterales, develops resistance to carbapenems due to the production of carbapenemases, porin alteration or loss, hyperexpression of efflux pumps, or alteration of PBP receptors [10]. Carbapenemases in *Proteus* spp. emerged twenty-seven years ago and belong to Classes A, B, and D.

### 7.1. Class A

Class A carbapenemases are rare in *Proteus* spp. The first report on KPC-2 harboring *P. mirabilis* was reported in the USA in 2008 [45] (Table 3). Later, a KPC-2-positive strain was identified as a causative agent of bloodstream infections in China [46], as illustrated in Table 3. The *bla*_KPC-2_ gene was located in IncN plasmid [46]. Antimicrobial susceptibility testing revealed that the strain was resistant to imipenem, meropenem, amoxicillin-clavulanic acid, ampicillin, ampicillin-sulbactam, cefotaxime, piperacillin, cefazolin, ciprofloxacin, levofloxacin, moxifloxacin, gentamicin, and sulfamethoxazole-trimethoprim but susceptible to ceftazidime, amikacin, aztreonam, and piperacillin-tazobactam. In keeping with its multidrug-resistant profile, *P. mirabilis* XH983 had a number of antimicrobial resistance genes (ARGs), conferring resistance to aminoglycosides (*aph*(3′)-Ia, *aph*(3″)-Ib, *aph*(6)-Id, *aac*(3)-IId, *aad*A5, and *aad*A1); β-lactam antibiotics (*bla*_KPC-2_ and *bla*_TEM-1B_); phenicol (*cat* and *cat*A1); sulfonamide-trimethoprim (*drf*A1, *drf*A17, *sul*1, and *sul*2); and tetracycline (*tet*(J)) [46].

### 7.2. Class B

VIM-1 (Verona integron-associated metallo-β-lactamase), which originates from *Pseudomonas aeruginosa*, was reported in *P. mirabilis* for the first time in 2006 in Greece [47] (Table 3). The isolate coproduced TEM-1, OXA-10, and ESBL VEB-1. It exhibited resistance to ESC and imipenem but remained susceptible to meropenem and aztreonam [47]. A later report from Bulgaria in 2019 described *P. mirabilis* isolates with chromosomally encoded VIM-1 carbapenemase, embedded in a class 1 integron, containing an IS*26* insertion element, aminoglycoside resistance genes *aac*(6)-Ib and *ant*(3)-1, and the *dfr*A1gene encoding dyhdropholate reductase, responsible for trimethoprim resistance. Increased resistance was related to the increased expression of the gene due to the increased gene copy number. The isolates showed variable resistance to carbapenems [48].

NDM was identified for the first time *in P. mirabilis* isolates from New Zealand in 2012 (Table 3). The isolate coharbored the *bla*_CTX-M-15_ gene and *rmt*C16SrRNA methylase gene for panaminoglycoside resistance [49]. In Europe, NDM was reported in *P. mirabilis* from Austria in combination with VEB-1 ESBL in 2018. The strain was imported from Bangladesh [34]. Recent studies showed the dissemination of NDM-producing *P. mirabilis* in animal settings among broilers in China [50]. The *bla*_NDM_ genes were carried either in plasmids or on chromosomes and, unlike humane isolates, exhibited high-level resistance to imipenem, meropenem, and ESC but remained susceptible to fluoroquinolones and aztreonam [50]. Inc A/C and Inc L/M are plasmid types responsible for the dissemination of *bla*_NDM_ genes worldwide.

IMP variants are the rarest [51]. IMP-1 and IMP-2 were found in *P. mirabilis* in Japan between 2000 and 2002. In the USA, there was a report on IMP-27. The *bla*_IMP-27_ gene is located in class 2 integrons. The isolates were recovered in different geographic areas of the USA but had similar resistance profiles, including resistance to ertapenem and susceptibility to piperacillin-tazobactam, ceftazidime, and aztreonam [51].

### 7.3. Class D

OXA-48 was originally described in *K. pneumoniae* in Turkey in 2004 [52] and, thereafter, has spread among different genera of *Enterobacterales* via highly conjugative IncL plasmids [53]. The first report of this widespread CHDL in *P. mirabilis* originated in 2015 from Russia [54], as shown in Table 3. Recently, OXA-48 with a very unusual resistance phenotype was described in nine *P. mirabilis* in Germany [55]. The isolates demonstrated susceptibility to imipenem and ertapenem and, in most cases, to piperacillin-tazobactam due to weak hydrolytic activity, which complicates laboratory detection and enables the isolates to be missed in routine diagnostic laboratories and create a hidden reservoir within hospitals, which is a source for dissemination of *bla*_OXA-48_ genes bypassing surveillance systems [55]. The *bla*_OXA-48_ genes in *P. mirabilis* were chromosomally encoded, and the gene was located on a genomic island, unlike those reported in other Enterobacterales, in which the gene resides in IncL plasmid and is surrounded by the IS*1999* insertion sequence upstream and downstream of the gene. In contrast to other *Enterobacterales*, mostly *E. coli* and *K. pneumoniae*, diffusion of the isolates was a consequence of the vertical transmission of related isolates and not the horizontal transfer of IncL plasmid, as in other *Enterobacterales*. Three isolates were found to harbor *bla*_OXA-181_ genes related to *bla*_OXA-48_, which were encoded in the IncX3 plasmid.

In France, 19 *P. mirabilis* isolates with slightly reduced susceptibility to carbapenems were analyzed, and OXA-23 CHDL was found, which is usually associated with *Acinetobacter baumannii*. The emergence of such a clone is worrisome, as it could be misidentified as penicillinase producers due to its susceptibility to carbapenems [56]. This enables these isolates to escape laboratory surveillance and to disseminate in the hospitals and community. All 19 isolates were clonally related but different from OXA-23 negative isolates. Moreover, a clonal lineage producing OXA-23 and OXA-58 was identified among 24 *P. mirabilis* strains from Belgium and France, including both human and animal isolates (Table 3) [57]. This indicates the spread of CHDL, which are typical for *A. baumannii* among *Enterobacterales*. OXA-23-encoding genes were located on the chromosome, while OXA-58 was plasmid-mediated [57]. The MICs of carbapenems were often in the susceptible range, and thus, the isolates were frequently not identified in the laboratory as carbapenemase producers, creating a potential reservoir for the spread of CHDL-encoding genes [57]. In addition to *bla*_OXA-23_, the strains harbored genes conferring resistance to aminoglycosides (*aph*(3″Ib and *aph*(6)-Id), sulphonamides (*sul*1 and *sul*2), trimethoprim (*dfr*A), and chloramphenicol resistance (*cat*). Unlike *A. baumannii*, the *bla*_OXA-23_ genes in *P. mirabilis* were not preceded by an IS*Aba1* element. This could explain very low carbapenem MICs [57]. The OXA-58-positive isolate originated from 1996, indicating the long-lasting undetected presence of CHDL in *P. mirabilis*. In Europe, except in France, OXA-58 was identified in *P. mirabilis* from Poland [58]. The β-lactam susceptibility pattern indicated resistance to penicillin (including temocillin). their β-lactamase inhibitor combinations; and carbapenems (with ertapenem, imipenem, and meropenem MICs of 8, 32, and 16 g/mL, respectively) and susceptibility to oxyimino compounds). The strain was resistant to fluoroquinolones and chloramphenicol and susceptible to amikacin, gentamicin, tobramycin, cotrimoxazole, and Fosfomycin [58].

**Figure 3:**
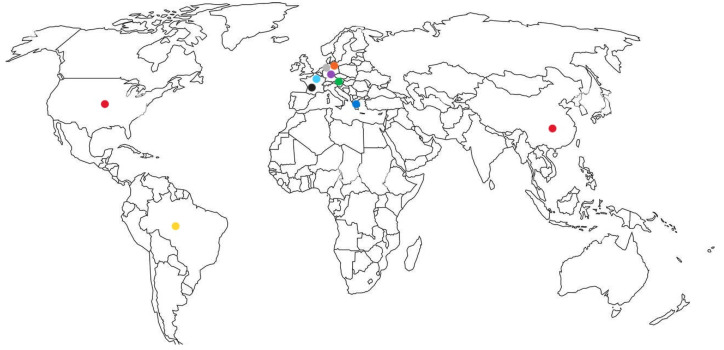
Distribution of carbapenemases in *Proteus* species. KPC-2, red; VIM-1, blue; NDM-5, green; KPC+NDM, yellow; OXA-48, orange; OXA-162, violet; OXA-181, gray; OXA-23, black; OXA-58, light blue.

**Figure 4:**
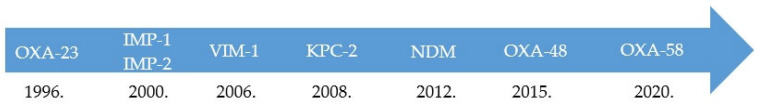
Flowchart showing the carbapenemase evolution in *Proteus* species.

The coproduction of double carbapenemases (KPC-2 and NDM-1) was reported in Brazil [59]. The isolates harbored a plethora of different virulence genes in addition to *bla*_KPC-2_, *bla*_NDM-1_, and *bla*_OXA-10_. Multiple carbapenemases are frequently encountered in *K. pneumoniae*, limiting therapeutic options [60]. The geographic distribution of carbapenemases is illustrated in Figure 3. The timeline of carbapenemases occurrence in *P. mirabilis* is presented in Figure 4.

**Table 3:** Most frequent carbapenemases in *Proteus* species.

Type of Carbapenemase	FEP	AMT	IMI	MEM	ERT	Geographic Distribution	References
KPC-2	R	R	R	R	R	USA, China	[45,46]
VIM-1	R	S	R	R	R	Greece	[47,48]
NDM-5	R	S	R	R	R	Austria	[34,49]
IMP-27	R	S	R	R	R	USA	[51]
KPC+NDM	R	R	R	R	R	Brazil	[59]
OXA-48	S	R	I-R	S	S	Germany, Russia	[54,55]
OXA-162	S	R	R	S	S	Germany	[54]
OXA-181	S	R	R	S	S	Germany	[54]
OXA-23	S	R	R	S	S	France Belgium	[56,57]
OXA-58	S	R	R (32)	R (16)	R (8)	France, Poland	[56,58]

Abbreviations: FEP—cefepime, AMT—aztreonam, IMI—imipenem, MEM—meropenem, ERT—ertapenem, R—resistant, I—intermediate susceptible (increased exposure), and S—susceptible. The MICs (mg/L) were provided in parentheses if available in the references.

## 8. Laboratory Detection of Extended-Spectrum β-Lactamases, Plasmid-Mediated AmpC β-Lactamases, and Carbapenemases in *Proteus* spp.

**Figure 5:**
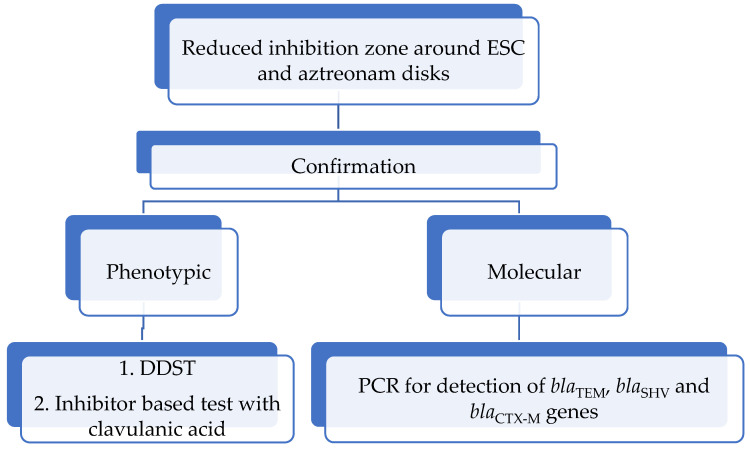
The algorithm for ESBL detection.

ESBLs are suspected based on the reduced susceptibility to ESC and aztreonam. The zone diameter size less than 22 mm for cefotaxime, 19 mm for ceftriaxone, 17 mm for ceftazidime and aztreonam, and 18 mm for cefepime is suspicious for ESBL positivity, according to the European Committee for Antimicrobial Susceptibility Testing (EUCAST) [61]. MIC values higher than 16 mg/L for ceftazidime and cefepime and above 4 mg/L for cefotaxime and ceftriaxone indicate resistance, which is most frequently associated with ESBL production, according to the Clinical Laboratory Standard Institution (CLSI) [62]. Confirmation of ESBLs in *Proteus* species is done by the double-disk synergy test (DDST), according to Jarlier [63], and combined disk test with clavulanic acid, according to the CLSI [62] (Table 4). The algorithm for ESBL detection is provided in Figure 5.

The augmentation of the inhibition zones of cephalosporin disks for at least 5 mm by clavulanic acid confirms ESBL production. Both methods have good sensitivity in *Proteus* spp. due to the lack of chromosomal AmpC β-lactamase, which could antagonize the synergistic effect with clavulanic acid (Table 4).

**Figure 6:**
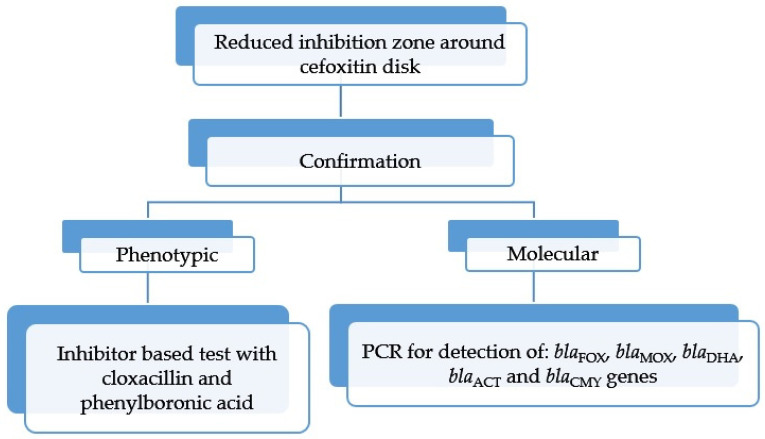
Algorithm for the detection of p-AmpC in *Proteus* species.

Screening for p-AmpC β-lactamases in *Proteus* spp. is based on reduced susceptibility to cefoxitin (zone diameter smaller than 19 mm (EUCAST) or MIC higher than 8 mg/L (CLSI)) [62]. Confirmation of p-AmpC is carried out by DDST, with a disk supplemented with 500 µg cloxacillin placed between the disks containing ceftazidime and cefotaxime on a lawn of *P. mirabilis* isolates with reduced susceptibility to cefoxitin in order to detect p-Amp-C [9] (Table 4). The distortion of the inhibition zones around ESC disks toward the central disk with cloxacillin was considered a positive result [9]. The other method for confirmation of P-AmpCs is an AmpC disk test, according to Black [64]. A blank paper disk is impregnated with 20 µL Tris-EDTA to permeabilize bacterial cells. Three to five colonies of the test organism are applied to the surface of the disk. The disk is placed on the surface of Mueller–Hinton (MH) agar previously inoculated with cefoxitin-susceptible *E. coli* ATCC 25922. The distortion of the inhibition zone around the cefoxitin disk indicated the enzymatic inactivation of cefoxitin [64]. An algorithm for p-AmpC detection is provided in Figure 6.

Isolates demonstrating reduced susceptibility to carbapenems, with zone sizes ≤ 19 mm for imipenem and meropenem and ≤18 mm for ertapenem or MIC values ≥ 4 mg/L against imipenem and meropenem and 2 mg/L against ertapenem [62], are subjected to screening for carbapenemase production by a modified Hodge test (MHT) [65], CIM (carbapenem-inactivation method) [66], eCIM [66], or CarbaNP test [67] (Table 4). Carbapenemase-positive isolates are tested for MBL positivity by the imipenem-EDTA inhibitor-based test [68].

For MHT, an overnight culture of carbapenem-susceptible indicator strain *E. coli* ATCC 25922 is inoculated on the surface of MacConkey agar plates to avoid swarming. After drying, an ertapenem disk (10 µg) is placed in the middle of the plate. Overnight, *Proteus* spp. cultures are streaked as a single line from the periphery of the disk to the edge of the plate. The plates are incubated overnight at 37 °C. Carbapenemase was suspected if the clover leaf indentation of the indicator organism was observed toward the ertapenem disk [65].

CIM is done to determine carbapenem hydrolysis. Overnight culture of the carbapenem-resistant test strain is suspended in saline, and an ertapenem disk (10 µg) is placed in the suspension, which was incubated for 2 h at 37 °C. As an indicator strain, *E. coli* ATCC 25922 is inoculated on Mueller–Hinton (MH) agar plates. The disk is removed after 2 h and placed in the middle of the plate. Carbapenem hydrolysis is confirmed if there is no inhibition zone, the zone is smaller than 14 mm, or if there are colonies within the inhibition zone [66].

The imipenem-EDTA inhibitor-based test is used to prove MBLs. Overnight, a *Proteus* spp. culture is spread on the MH agar plate. Imipenem and meropenem disks with and without EDTA are placed on the plate. Cultures are incubated overnight at 37 °C. The augmentation of the inhibition zone around the carbapenem disk for at least 7 mm in the presence of EDTA is considered a positive result [68].

CHDL detection in *Proteus* spp. poses a challenge for microbiology laboratories because of very low MICs and large inhibition zones around carbapenem disks, which are often in the susceptibility or intermediate susceptibility (increased exposure) range. Moreover, ceftazidime, cefepime, and piperacillin-tazobactam are susceptible in the majority of isolates. There is a new algorithm for the detection of OXA-48 in *Proteus*, proposed by Hamprecht et al. [69]. OXA-48 is suspected based on a resistance to temocillin, which is not hydrolyzed by Class A or B carbapenemases. The next step is testing of the hydrolysis of the meropenem substrate by mCIM test and eCIM. Multiplex PCR or immunochromatographic tests are recommended to determine the type of carbapenemase. The *bla*_OXA-23_ and *bla*_OXA-58_ genes should be included in the panel for molecular testing, in addition to the *bla*_KPC_, *bla*_IMP_, *bla*_VIM_, *bla*_NDM_, and *bla*_OXA-48_ genes [70] (Table 4). Accurate and fast identification of resistance determinants should be mandatory for proper antibiotic choice and for epidemiologic reasons to limit the spread of the isolates harboring resistance traits. French authors proposed a screening method for OXA-23-producing organisms based on reduced susceptibility to amoxicillin-clavulanate (<11 mm) [71]. Confirmation is usually done by immunochromatographic tests or PCR. Moreover, the isolates producing OXA-48 carbapenemase often show susceptibility to cephalosporins, unless they possess an additional ESBL, and thus, they are not recognized as multidrug-resistant strains. For epidemiology reasons, it is important to determine if the resistance genes are carried on bacterial chromosomes or plasmids. Plasmids are characterized based on their incompatibility groups, which are characterized by PCR-based replicon typing, according to Carattoli et al. [72]. The procedure is performed with eighteen pairs of primers, including five multiplex and three single-plex PCRs, which reveal the plasmid incompatibility group (Inc). An updated method is now available to determine the IncL plasmid, which usually carries *bla*_OXA-48_ genes [73].

**Table 4:** Tests for phenotypic and molecular detection of beta-lactamases among Proteus species isolates.

Resistance Determinant	Screening	Phenotypic Confirmatory Tests	Molecular Tests
ESBL	Reduced inhibition zones around ESC and aztreonam	DDST Combined disk test with clavulanic acid	PCR for *bla*_TEM_, *bla*_SHV_, *bl*a_CTX-M_, and *bla*_PER_ genes
p-AmpC	Reduced inhibition zones around cefoxitin disk	combined disk test with cloxacillin or phenyboronic acid	PCR for *bla*_FOX_, *bla*_MOX_, *bla*_DHA_, *bla*_CMY_, and *bl*a_ATC_ genes
carbapenemase	Reduced inhibition zones around imipenem, meropenem or ertapenem disk	Modified Hodge test, CIM, eCIM, CarbaNP test	PCR for *bla*_KPC_, *bla*_IMP_, *bla*_VIM_, *bla*_NDM_, and *bla*_OXA-48_ genes

## 9. Clinical Significance of Multidrug-Resistant (MDR) and Extensively Drug-Resistant (XDR) Isolates

The data on the clinical outcome of severe infections with multidrug-resistant *Proteus* spp. are scarce in the medical bibliography. The species most frequently involved in severe infections like sepsis are *P. mirabilis*, *P. vulgaris*, and *Proteus penneri* [74]. An Italian study showed that ESBL positivity increases the risk of therapeutic failure and poor outcome in the case of bloodstream infections (BSIs) [75]. Twenty-five BSI episodes caused by *P*. *mirabilis* were analyzed. Eleven isolates were found to produce ESBL belonging to the TEM family (TEM-52 or TEM-92). Previous hospitalization, stay in a long-term care facility, and the presence of urinary catheters were risk factors for acquisition of an ESBL strain. BSI cases due to ESBL-negative isolates uniformly responded to therapy, whereas 5/11 cases due to ESBL-positive isolates failed to respond (*p* < 0.01). the use of carbapenems was associated with a complete response independently of ESBL production. Therapeutic failure and mortality may occur in BSI episodes caused by ESBL-positive *P*. *mirabilis* isolates. Thus, the recognition of ESBL-positive strains appears to be critical for clinical management.

A study carried out in Australia [76] found that *Proteus* species BSIs occur at an incidence rate of 2.8 per 100,000 population annually and that elderly males are at the highest risk. *Proteus* species comprise approximately 1.7% of BSI isolates overall, and 4.1% of BSIs due to Enterobacterales in the study population. Given their occurrence, the median length of stay of ≥10 days, and that one in five patients die within 30 days of diagnosis, it is evident that *Proteus* species BSIs are associated with a substantial disease burden.

There are no bibliographical data on severe infections with AmpC- or carbapenemase-producing *P. mirabilis*.

Recently, six extensively drug-resistant (XDR) isolates producing ESBLs, p-AmpC, and carbapenemases were identified in Egypt [77]. The isolates produced OXA-48 or NDM carbapenemase, in addition to p-AmpC and ESBL belonging to the CTX-M-family, and gene cassettes containing aminoglycoside resistance genes (*aad*A2 and *aad*B), dihydrofolate reductase genes (*dfr*A17), and the *bla*_OXA-10_ gene embedded in a class 1 integron.

## 10. Therapeutic Options

From a therapeutic point of view, it is important to distinguish between ESBLs and AmpC β-lactamases among ESC-resistant isolates, because infections caused by AmpC-positive isolates can be effectively treated with cefepime and cefpirome. On the other hand, uncomplicated urinary tract infections due to ESBL-positive organisms can be treated with β-lactam/inhibitor combinations, which are not recommended for AmpC-producing organisms [78]. The CLSI recommends all ESC to be reported as resistant if the isolate produces an ESBL, regardless of the in vitro susceptibility results, to avoid therapeutic failures [62]. Even if the cephalosporin is active in vitro, during therapy, mutants hyperproducing ESBLs can develop and cause therapeutic failures. According to EUCAST, in the case of ESBL positivity, the results are reported as they are but with the recommendation to use carbapenems in cases of serious, life-threatening infections [61].

On the other hand, CLSI has yet to establish a testing and reporting algorithm specifically for organisms containing AmpC β-lactamases. The identification of AmpC β-lactamases in *E. coli*, *P. mirabilis*, and *Klebsiella* spp. can increase the accuracy of antimicrobial testing reports for expanded-spectrum cephalosporins if the results are used to modify the interpretations of cephalosporin results. Some studies have shown comparable or equal activity of cefepime and piperacillin-tazobactam against AmpC-producing Enterobacterales [79,80].

Carbapenem-resistant isolates of *P. mirabilis* pose a serious therapeutic challenge due to the intrinsic resistance to colistin, nitrofurantoin, and tigecycline, which limits the therapeutic options. There are now new therapeutic options available—in particular, novel β-lactam–inhibitor combinations. Ceftazidime-avibactam demonstrates excellent activity against KPC- and OXA-48-producing organisms but is not active on stains positive for MBLs [81,82,83]. Ceftolozane-tazobactam has good activity against ESBL-positive strains and those with porin loss or alteration and hyperexpression of efflux pumps but does not exert activity on carbapenemase-positive isolates. Imipenem-cilastatin-relelbactam and meropenem-varbobactam inhibit isolates with KPC and OXA-48 but do not exert activity on MBL-producing organisms (Table 5). The last line of therapy for infections associated with MBL-producing isolates is cefiderocol [83]. The rate of susceptibility to ceftazidim-avibactam in *Proteus* species is 100%, to ceftolozane-tazobactam is 89%, and to meropenem-varbobactam is 99% [84].

Cefiderocol (FDC) is the first-in-class catechol-siderophore-cephalosporin approved in the European Union (EU) with potent activity against carbapenemase-producing *Enterobacterales* [85] (Table 5). FDC binds to penicillin-binding proteins (PBPs) to block the final stage of the peptidoglycan synthesis [86]. Its activity is not compromised by upregulation of efflux pumps or porin alteration [86]. However, there have been increasing reports indicating correlations between the production of carbapenemases and reduced susceptibility to FDC [86]. The activity of FDC is decreased in the presence of MBLs, particularly NDM, and ESBLs belonging to PER-1. There has been no resistance to cefiderocol among *P. mirabilis* isolates reported so far [86].

## 11. Conclusions

This review demonstrated the amazing capacity of *Proteus* species to acquire various resistance determinants in addition to intrinsic resistance and to develop multidrug or extensively drug resistance phenotypes with a few or no therapeutic options left. Accurate and fast laboratory identification of resistance determinants is mandatory to avoid the spread of resistance isolates and hospital outbreaks. Confirmation of the genes encoding ESBLs, AmpC β- lactamases, and carbapenemases is of high epidemiological relevance in order to choose the appropriate therapy for bacterial infections due to multidrug-resistant *Proteus* species. The same allelic variants of ESBL and p-AmpC genes were found in both human and animal isolates from different geographic areas and continents, reinforcing the “One Health” approach. CTX-M-3, CTX-M-15, and CTX-M-65 are shared between the humans and animals [87], and among the carbapenemases, OXA-23 and OXA-58 [56] are reported in both settings. The dominant animal species harboring ESBL and AmpC-positive *P. mirabilis* are broilers, which are food-producing animals and can serve as a source of human intestinal colonization. *P. mirabilis* is an important host organism for CHDL previously identified in *A. baumannii* such as OXA-23 and OXA-58, unlike other Enterobacterales, but with much weaker expression and developing clinically relevant resistance only in the presence of other resistance determinants like porin loss or hyperexpression of efflux systems. There is no need for the implementation of hospital hygiene measures in cases of CHDL among *Proteus* spp., because the isolates are susceptible to the majority of antibiotics, but the clinical–laboratory correlations have not been investigated yet. Moreover, it took over genes encoding rare ESBL types such as PER-1 or VEB-1, rarely encountered among *Enterobacterales*, probably from *P. aeruginosa*. Laboratory identification of carbapenemases poses a serious challenge for clinical microbiologists because of the very low MICs of carbapenems alongside the susceptibility to ceftazidime, cefepime, and piperacillin-tazobactam, resulting in the high risk of carbapenemases being overlooked in the laboratory. For that reason, a new algorithm was proposed for the detection of carbapenemases in *Proteus* species. The emergence and spread of carbapenemase-producing *P. mirabilis* is an important threat to the patients and a challenge for clinicians. BSIs caused by ESBL-positive organisms have significantly higher mortality rates compared to ESBL-negative isolates. New β-lactam–inhibitor combinations and cefiderocol have promising activity against resistant isolates.

## Figures and Tables

**Table 2:** Most frequent AmpC β-lactamases in *Proteus* species.

	Resistance Phenotype							
Type of AmpC	CAZ	CTX	CRO	FEP	AMT	FOX	Geographic Distribution	References
CMY-16	R (>128)	R (>128)	R (>128)	S	R	R (>128)	Croatia Italy	[39,40,41]
CMY-2	R	R	R	S	R	R	Italy Taiwan Brazil	[37,42,43]
DHA-1	R	R	R	S	R	R	Poland	[44]

Abbreviations: CAZ—ceftazidime, CTX—cefotaxime, CRO—ceftriaxone, FEP—cefepime, AMT—aztreonam, FOX—cefoxitin, R—resistant, and S—susceptible. The MICs (mg/L) were provided in parentheses if available in the references.

**Table 5:** Activity of β-lactam novel antibiotics against carbapenemase-producing *Proteus* spp.

	ESBL	KPC	MBL (IMP, VIM, NDM)	OXA-48
Ceftazidime-avibactam	+	+	−	+
Ceftolozane-tazobactam	+	−	−	−
Imipenem-relebactam	+	+	−	+
Meropenem-varbobactam	+	+	−	+
Cefiderocol	+	+	+	+

+ compound is active; − compound is not active

## Data Availability

No new data were created or analyzed in this study.

## Abbreviations

ESBL	extended-spectrum β-lactamases
p-AmpC	plasmid-mediated AmpC β-lactamases,
ESC	expanded-spectrum cephalosporins
CHDL	carbapenem-hydrolyzing class D oxacillinases
MIC	minimum inhibitory concentration
WGS	whole genome sequencing
PFGE	pulsed-field gel electrophoresis
BSI	bloodstream infection
CLSI	Clinical Laboratory Standard Institution
EUCAST	European Committee for Antimicrobial Susceptibility Testing
MHA	Mueller-Hinton agar
OKNV	OXA-48, KPC, NDM, VIM
CAZ	ceftazidime
FEP	cefepime
CTX	cefotaxime
CRO	ceftriaxone
AMT	aztreonam
IMI	imipenem
MEM	meropenem
ERT	ertapenem
MIC	minimum-inhibitory concentration
